# Comparison of NK alloreactivity prediction models based on KIR-MHC interactions in haematopoietic stem cell transplantation

**DOI:** 10.3389/fimmu.2023.1028162

**Published:** 2023-03-02

**Authors:** Adèle Dhuyser, Thomas Remen, Michaël Pérès, Vitalina Chamberlain-Evans, Neda Nemat-Gorgani, Arnaud Campidelli, Sandra Clément, Marie Thérèse Rubio, John Trowsdale, Alice Aarnink, James Traherne

**Affiliations:** ^1^Histocompatibility Laboratory, Centre Hospitalier Régional Universitaire de Nancy, Nancy, France; ^2^Ingénieurie Moléculaire et Physiopathologie Articulaire, team 6 (IMoPA6), Unité Mixte de Recherche 7365 Centre national de la Recherche Scientifique, Université de Lorraine, Nancy, France; ^3^Direction de la Recherche Clinique et de l’Innovation, Unité de Méthodologie, Datamanagement et Statistiques, Centre Hospitalier Régional Universitaire de Nancy, Nancy, France; ^4^Department of Pathology, University of Cambridge, Cambridge, United Kingdom; ^5^Department of Hematology, Centre Hospitalier Régional Universitaire de Nancy, Nancy, France

**Keywords:** KIR, MHC, HLA, alloreactivity, prediction, natural killer (NK), allogeneic stem cell transplantation, donor selection

## Abstract

The biological processes underlying NK cell alloreactivity in haematopoietic stem cell transplantation (HSCT) remain unclear. Many different models to predict NK alloreactivity through KIR and MHC genotyping exist, raising ambiguities in its utility and application for clinicians. We assessed 27 predictive models, broadly divided into six categories of alloreactivity prediction: ligand-ligand, receptor-ligand, educational, KIR haplotype-based, KIR matching and KIR allelic polymorphism. The models were applied to 78 NGS-typed donor/recipient pairs undergoing allogeneic HSCT in genoidentical (n=43) or haploidentical (n=35) matchings. Correlations between different predictive models differed widely, suggesting that the choice of the model in predicting NK alloreactivity matters. For example, two broadly used models, *educational* and *receptor-ligand*, led to opposing predictions especially in the genoidentical cohort. Correlations also depended on the matching fashion, suggesting that this parameter should also be taken into account in the choice of the scoring strategy. The number of centromeric B-motifs was the only model strongly correlated with the incidence of acute graft-versus-host disease in our set of patients in both the genoidentical and the haploidentical cohorts, suggesting that KIR-based alloreactivity, not MHC mismatches, are responsible for it. To our best knowledge, this paper is the first to experimentally compare NK alloreactivity prediction models within a cohort of genoidentical and haploidentical donor-recipient pairs. This study helps to resolve current discrepancies in KIR-based alloreactivity predictions and highlights the need for deeper consideration of the models used in clinical studies as well as in medical practice.

## Introduction

Allogeneic haematopoietic stem cell transplantation (aHSCT) is an immunotherapy based on donor cell alloreactivity. This procedure can be used in a wide spectrum of diseases but especially as standard of care in haematological malignancies, when chemotherapies are insufficient for tumorigenesis control (1). Indeed, alloreactivity mediated by the donor’s competent cells contained within the graft can achieve a life-saving graft versus leukemia (GVL) effect, where the graft destroys leukemic cells. However, alloreactivity also risks life-threatening effects such as graft versus host disease (GVHD).

The limited availability of 10/10 HLA-matched donor, either from sibling or unrelated donors, has restricted aHSCT feasibility for decades. However, current data are consistent with good outcomes using haploidentical donors (2). Together with favourable practical aspects to their use, this led to a 291% increase of haploidentical aHSCT in Europe between 2005 and 2015 (3) and a further 11% between 2018 and 2019 according to the last EBMT activity survey report (4).

Considering that at least one first-degree relative haploidentical donor is available for 95% of patients with haematological malignancies (5), the use of haploidentical donors leads to a major paradigm shift: while donor availability represented the main drawback for years, the issue now becomes one of finding the best haploidentical donor among several potential candidates. A major focus of attention is the assessment of natural killer (NK) cell alloreactivity through Killer Immunoglobulin-like Receptor (KIR) genotyping.

NK cells are innate lymphoid cells involved in early immunity against tumors (6). Their cytolytic activity is finely regulated to prevent inappropriate activation against normal cells through a process called “education” in which the interaction between KIRs and their cognate ligands appears to be crucial (7, 8). This process is not fully understood but the current consensus requires an NK cell to interact with a major histocompatibility complex (MHC) class I molecule *via* one of its KIR receptors to become competent, i.e. to be able to activate itself against abnormal cells (9). NK cells that have not had any contact with an MHC class I molecule remain inactive and thus less able to cause damage by autoreactivity. According to the missing-self theory, each competent NK cell maintains self-tolerance to autologous healthy cells, while it can recognize and kill abnormal cells that have downregulated MHC class I molecules (10).

In context of aHSCT, there is general agreement that NK cells are important players in the GVL effect while alloreactive TCRαβ T-lymphocytes are mainly responsible for GVHD. Indeed, kinetics of recovery and rates of early post-transplant NK cells reached during the period of severe lymphopenia correlate with lower relapse rates and better survival (11, 12), suggesting that this alloreactivity could be responsible for an early crucial GVL effect. Moreover, the historic functional study led by Ruggeri et al. has clearly shown that alloreactivity directly mediated by NK cells does not increase the rate of GVHD (13). Shah et al. have however reported that the adoptive transfer of donor-derived activated NK cells following HLA-matched T-cell depleted aHSCT led to especially high rates of GVHD: five of nine transplant recipient experienced GVHD, with grade 4 GVHD observed in 3 subjects (14). Given that the T-cell dose was below the threshold required for GVHD in this setting, Shah et al. conclude that activated donor’s NK indirectly contributed to the acute GVHD (aGVHD) observed, likely by augmenting underlying T-cell alloreactivity, which is consistent with murine observations (15).

Finally, clinical studies remain discrepant regarding the association of NK predicted alloreactivity and positive or negative allograft outcomes (16). Problems in assessing these data include: (i) the clinical studies are mainly retrospective and the data collection partial (ii) some studies span periods up to 20 years over which time standards of care and technologies for MHC/KIR assessments have greatly evolved (iii) heterogeneity of cohorts especially in terms of ethnicities, pathologies (myeloid vs lymphoid malignancies) and ages (paediatric vs adult populations) (iv) heterogeneity of graft procedures leading to various quantity and quality of NK cells (11, 17, 18).

Besides these possible explanations, heterogeneous KIR-MHC based models have been used to predict NK alloreactivity, which could also be partly responsible for discrepancies. Therefore, the objective of the current work was to compare the different approaches predicting NK alloreactivity. We also explored correlations between the models with the following clinical outcomes: death, relapse, aGVHD and chronic GVHD (cGVHD).

## Materials and methods

### Cohorts

Two independent cohorts of patients undergoing aHSCT and their respective siblings – genoidentical for the first cohort and haploidentical for the second - were compiled in this study. The genoidentical cohort includes 43 donor/recipient (D/R) pairs for whom dried pellets derived from peripheral blood and clinical data were provided by the CRYOSTEM consortium (https://doi.org/10.25718/cryostem-collection/2018) and the SFGM-TC. The haploidentical cohort includes the 35 first consecutive haploidentical D/R pairs belonging to the local biocollection established by the histocompatibility laboratory at Centre Hospitalier Régional Universitaire of Nancy, France. More details are to found in **Supplementary Data**, section “Materials and methods”.

This noninterventional research study is registered to the Protocol Registration and Results System under the number NCT04882605. It has been approved by the local ethic committee and carried out in accordance with the current French and European ethical standards, as well as the code of ethics of the World Medical Association. All patients gave their written consent for the generation and use of genetic data for research purposes as well as clinical data collection, not allowing the identification of individual subjects in accordance with the MR-004 reference edited by the Commission Nationale Informatique et Liberté (number 2020PI142-173 in the register of CNIL activities of the CHRU of Nancy).

### MHC and KIR genotyping

DKMS Life Science Lab GmbH provided allelic genotyping resolution of all 13 KIR genes (*(KIR2DL1, KIR2DL2/3, KIR2DL4, KIR2DL5A, KIR2DL5B, KIR2DS1, KIR2DS2, KIR2DS3, KIR2DS4,KIR2DS5, KIR3DL1/3DS1, KIR3DL2, KIR3DL3)*, 2 KIR pseudogenes (*KIR2DP1* and *-3DP1*) and HLA genes (*HLA-A, -B, -C, -DRB1, -DQA1, -DQB1, -DPA1, -DPB1* and *-DRB3/4/5)* for all enrolled individuals as previously described (19, 20). For the haploidentical pairs, presence or absence of donor’s KIR genes and pseudogenes was also assessed by PCR-SSO using the Luminex^®^ xMAP^®^ technology.

To avoid artefacts (e.g. DNA contamination or sample inverting) the linkage disequilibria between HLA-B/-C and HLA-DR/-DQ (21) as well as the known KIR copy numbers imbalances (22) were verified as described previously, plus the consistency of HLA compatibility within each D/R couple.

Details about DNA extraction and MHC/KIR genotyping can be found in **Supplementary Data**, section “Material and methods”.

### Alloreactivity prediction

**Figure 1:**
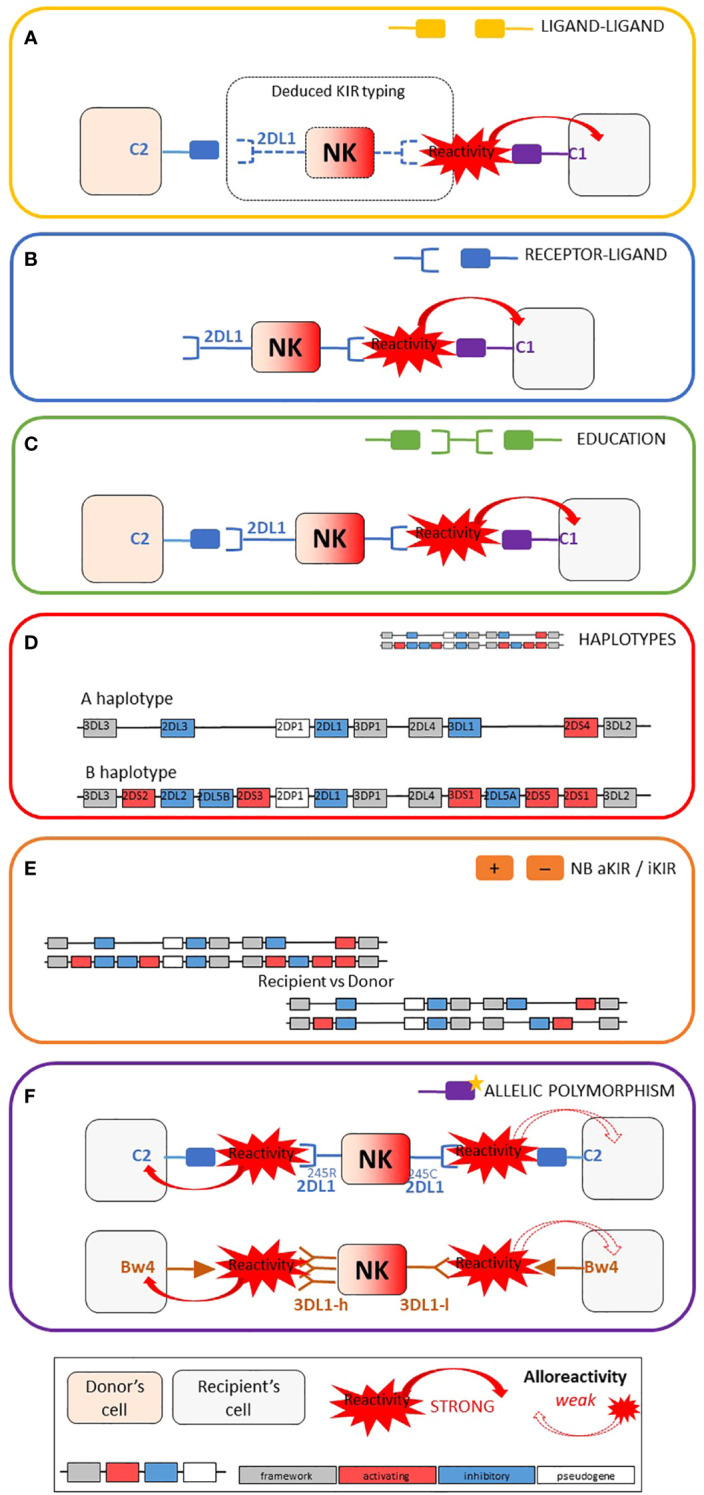
Schematic representation of the six main categories of models used for NK alloreactivity prediction and summary table of the characteristics of each category. Adapted from Dhuyser et al. (16). **(A)** Ligand–ligand model confronts the MHC of the donor with the MHC of the recipient: KIR genotyping is unknown and NK alloreactivity of the donor toward host cells is expected when the recipient lacks MHC class I ligand present in the donor. **(B)** Receptor–ligand model considers the KIR of the donor and the MHC of the recipient: if at least one KIR gene expressed in the donor does not recognize any of the MHC molecules of the recipient (“missing-ligand”), the NK cells of the donor will increase their cytotoxic activity. **(C)** Educational models consider the MHC class I molecules of the donor and recipient and the KIR typing of the donor. It should reflect the “education” process required for NK cells to become competent. **(D)** The KIR haplotypes of the donor: the B/x of the donor and particularly those carrying Cen-B/B are expected to be more alloreactive toward the cell of the recipients, as they carry mostly activating KIR genes. **(E)** KIR matching models represent the number of aKIR and/or iKIR gene present in the donor but absent in the recipient and vice versa. **(F)** KIR polymorphism leads to KIR molecules with relevant biological differences.

**Figure 2:**
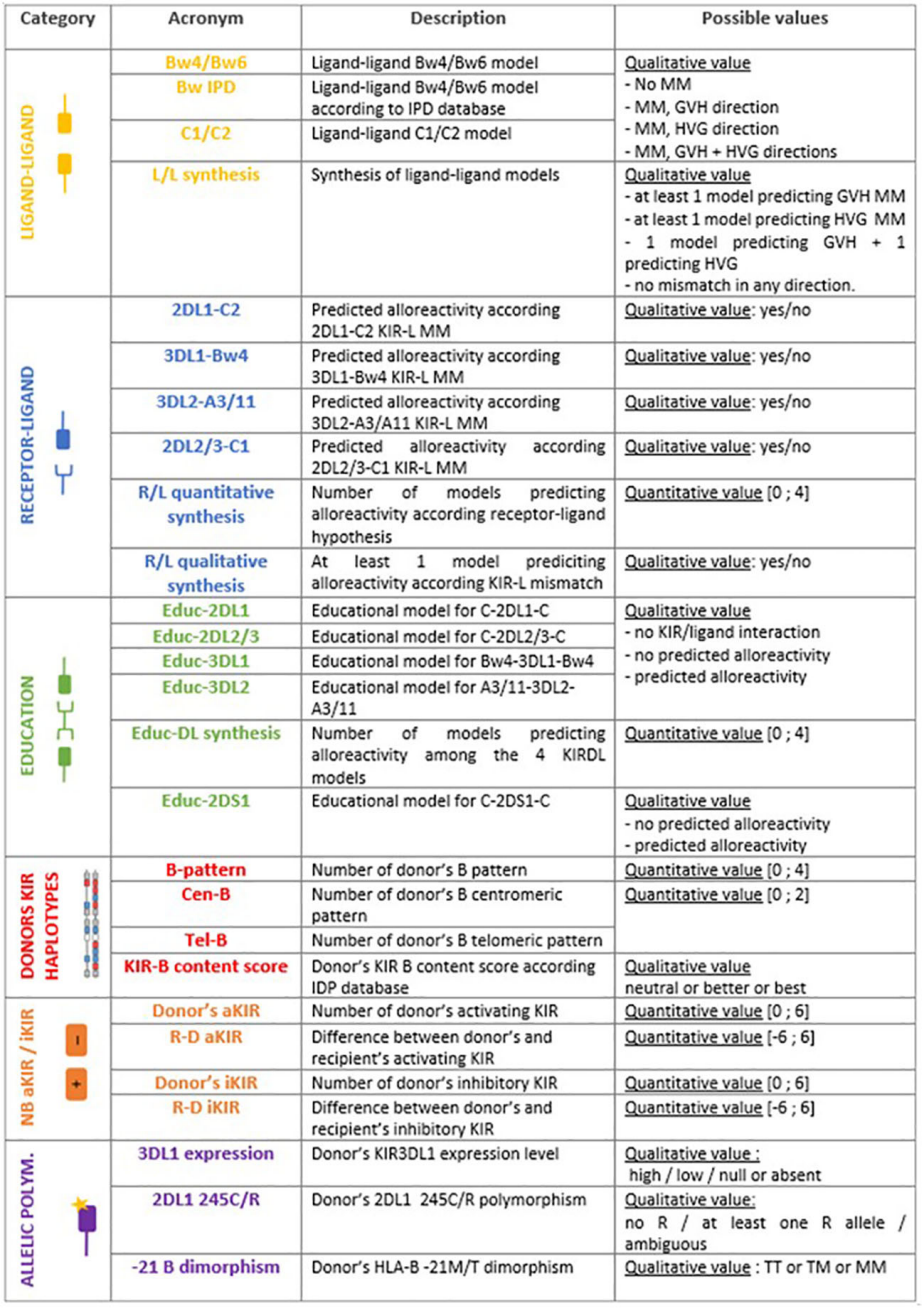
Acronyms used in this paper, short description of each model and possible values of alloreactivity assessment.

**Figures 1A–F** represent the six main categories of NK cell alloreactivity prediction models that have been applied to our two cohorts: 24 models divided into five categories of alloreactivity prediction based on KIR presence or absence (ligand-ligand, receptor-ligand, educational, KIR haplotype-based and KIR matching) as well as three models based on KIR allelic polymorphism. **Figure 2** also shows the acronyms for each of the 27 models used in the rest of this article, as well as a brief description and the possible values according to our research methodology.

#### Ligand-ligand models

Donor NK cells are expected to be alloreactive toward host cells when the recipient is lacking MHC class I ligand present in donor (18). This drives the beneficial GVL effect as well as its GVHD counterpart. Conversely, mismatch in host versus graft (HVG) direction is assumed when MHC class I ligand is present in recipient but not in donor. In the Bw4/Bw6 model, HLA-Bw4/-Bw6 epitopes were manually annotated according to the amino acid at positions 77 and 80 of α1 domain of HLA class I heavy chain, but also taking into account the Bw4 and Bw6 epitopes that might be carried by certain HLA-A or HLA-C molecules respectively.

In the same fashion, HLA-C1/-C2 epitopes were manually annotated according to amino acid at position 80 of α1 domain of HLA class I heavy chain to assess the C1/C2 model.

A D/R couple can therefore be either matched, mismatched in GVH direction or mismatched in HVG direction for HLA-B epitopes (Bw4/Bw6) and/or HLA-C epitopes (C1/C2).

The IPD database proposes online assessments of mismatches by entering the D/R couple HLA-B and -C typing (23) that we used through the BwIPD model. Unlike the manual annotation above, this algorithm does not take into account the Bw4 and Bw6 epitopes that are carried by certain HLA-A or HLA-C molecules, respectively.

Ligand-ligand synthesis aims to assess ligand-ligand models across HLA-B and HLA-C so the D/R pairs fall into one of four categories; (i) at least one model predicting mismatch in the GVH direction, (ii) at least one model predicting mismatch in the HVG direction, (iii) one model predicting mismatch in the GVH direction whereas the second model is predicting mismatch in the HVG direction, or (iv) no mismatch in any direction.

#### Receptor-ligand/missing-ligand models

Donor NK cells are expected to be alloreactive toward host cells when the donor possesses inhibitory KIR for which the recipient lacks ligand (24). Accordingly, the following models: 2DL1-C2, 3DL1-Bw4, 3DL2-A3/11 and 2DL2/3-C1 reflect the receptor-ligand mismatch for each known inhibitory KIR/MHC interaction pair.

The synthesis of these receptor-ligand mismatch models was done in a quantitative way - the number of missing-ligand models predicting an alloreactivity (R/L quantitative synthesis) - and in a qualitative way - at least one missing-ligand model predicting an alloreactivity (R/L qualitative synthesis).

#### Education models

**Figure 3:**
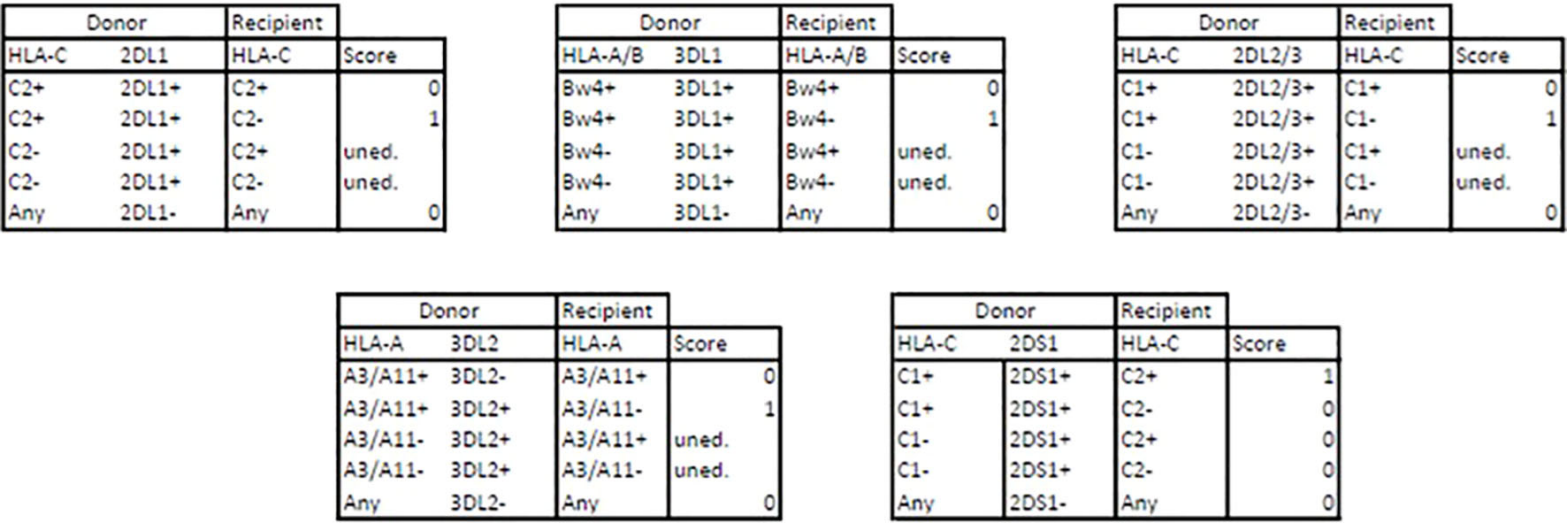
Alloreactivity assessments through educational models. D, donor; R, recipient; 0, no alloreactivity; 1, predicted alloreactivity ; uned., uneducated.

As a reflection of the education process required for a NK cell to become fully functional and reactive, the KIR mismatch leading to alloreactivity was assessed when the donor has licenced NK cells - i.e. inhibitory KIR and its cognate MHC ligand - but the recipient lacks the cognate MHC ligand (25, 26). Thus, the donor’s NK cells either show (i) no alloreactivity because they lack certain KIR or D/R are matched for cognate MHC, (ii) remain uneducated because the donor does not have MHC ligand or (iii) are predicted to be educated and alloreactive for each model (Educ-2DL1, Educ-2DL2/3, Educ-3DL1 and Educ-3DL2) as summarized in the **Figure 3**. It is worth to note that each KIR haplotype carries a *KIR3DL2* gene since it is a framework gene, and a version of either *KIR2DL2* or *KIR2DL3* gene as these are alleles of the same *KIR2DL2/3* gene, carried by either the B-haplotype or the A-haplotype, respectively.

Educ-DL synthesis summarizes the number of Educ_xDLx models that predict alloreactivity among the four previously described KIRxDLx models.

There is also a potential educational process achieved by the activating KIR2DS1 and its cognate C2 ligand (27). Donor 2DS1+ C1+ NK cells are fully educated and capable of recognizing their ligands on recipient’s C2+ leukemia cells. Conversely, donor 2DS1+ C1- NK cells remain hyporesponsive regardless of the ligands exhibited by leukemic cells (28). This educational process is described through the Educ-2DS1 model.

#### KIR haplotype based models

KIR genes are in strong linkage disequilibrium and are transmitted in haplotypes (29). Two main haplotypes have been described: A-haplotypes mostly contain inhibitory KIR (iKIR) whereas B-haplotypes contain one or more activating KIR (aKIR) gene. Each of these haplotypes can be divided into a centromeric (Cen) and a telomeric (Tel) part. Many studies have focused on the contribution of each particular motif of donors’ KIR haplotypes (e.g. Cen-A, Cen-B, Tel-A, Tel-B, AA-haplotypes vs Bx haplotypes) in the generation of alloreactivity. We assessed the following scores counting the number of donor Cen-B motifs, Tel-B motifs, and number of donor B-motifs either Cen or Tel (B-pattern). As a synthesis of the previous models, Cooley et al. have designed the KIR-B content score that considers both the centromeric and telomeric parts in the assessment of alloreactivity (30). KIR-B content score can be generated by entering the donor KIR type (presence or absence of each gene) on the IPD database website (31), classifying the donors as “neutral”, “better” or “best”.

#### Gene content models and gene-gene models

T the number of donor aKIR and donor iKIR can be assessed, as well as the differences between donor and recipient numbers of aKIR and iKIR respectively through R-D aKIR and R-D iKIR models. Framework iKIR were excluded from counting.

#### Allelic polymorphism

KIR3DL1/S1 allele products have been correlated with high (KIR3DL1-h) or low (KIR3DL1-l) cell-surface densities. They may also either be retained within the cell (KIR3DL1-n) or displayed on the cell surface but do not bind HLA-Bw4 (KIR3DS1). Some studies correlate KIR3DL1-h and Bw4-80I with stronger NK inhibition and a reduced alloreactivity (32, 33). The 3DL1 expression model explores donor KIR3DL1 expression as described by Boudreau et al. (33). When the donor had two alleles with different expression level, the highest level of expression was retained. The association with Bw4 has not been tested because of the small cohort size.

KIR2DL1 receptors can be split in two groups according to the amino acid at position 245 of its transmembrane domain: KIR2DL1-R245 and KIR2DL1-C245 with arginine or cysteine, respectively. As KIR2DL1-R245 is known to signal stronger than KIR2DL1-C245, we explored this dimorphism using the 2DL1 245C/R model, that split the cohorts into two groups considering the presence or absence of at least one KIR2DL1-R245 receptor, assessed as described by Bari et al. (34).

KIR are not the only receptors responsible for NK alloreactivity*. HLA-B* dimorphism at position −21 (methionine or threonine, thus M/T dimorphism) in the segment encoding the leader peptide dictates whether NK cell regulation primarily relies on the KIR or another important heterodimer receptor, NKG2A/CD94. Subjects carrying HLA-B -21M harbor better-educated NKG2A^+^ NK cells and display superior capacity to degranulate lytic granules against KIR ligand-matched primary leukemic blasts (35). Donor’s HLA-B 21M/T dimorphism was therefore explored using the -21B dimorphism model.

### Data collection

Relapse was defined by the reappearance of malignant cells in the blood smear, the loss of molecular or cytogenic remission for myeloid neoplasia, or the reappearance of radiological lesions for lymphoid neoplasia. We assessed aGVHD and cGVHD as defined by international consensus (36, 37). aGVHD was considered only from grade II according to the Glücksberg classification. More details are to be found in **Supplementary Data**, section “Materials and methods”.

### Statistical analysis

Categorical variables are described by their counts and percentages, and continuous variables by their mean and standard deviation or median and extreme values depending on the variable’s distribution. The comparison of patients’ characteristics between the two groups (genoidentical vs haploidentical) was carried out through Chi-2 tests or Fisher’s exact tests for the categorical variables and by Student’s or Mann-Whitney U tests for the continuous variables. The strength of the association between the different biological scores was explored through Spearman’s correlation coefficients. The comparison of the cumulative incidence of the various events according to the results of biological scores was carried out using the Gray test (survival model), death being considered as a competitive risk in the various models.

## Results

### Distribution of the predicted alloreactivity according to the models

**Figure 4:**
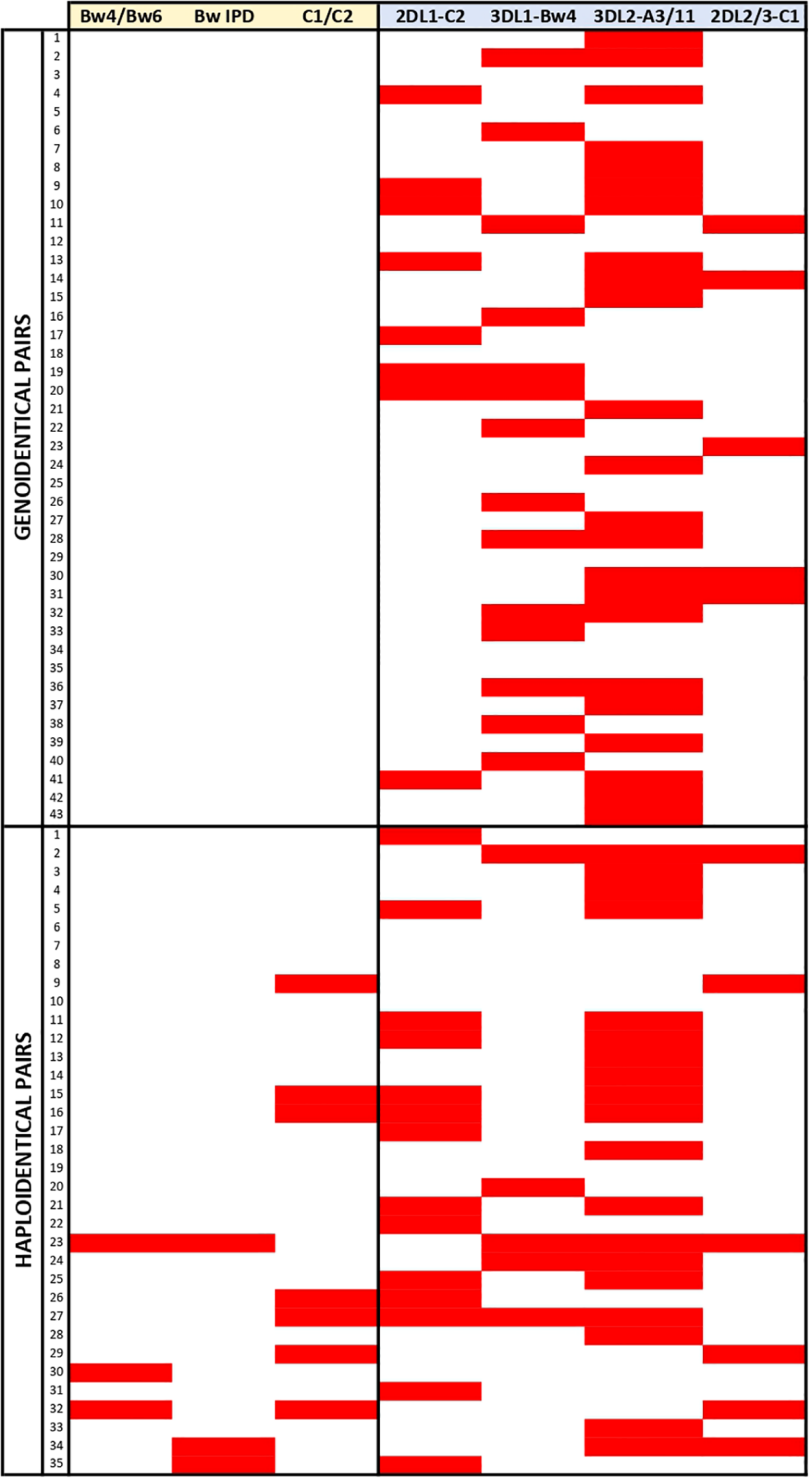
Representation of the alloreactivity prediction according the models of the ligand/ligand and receptor/ligand categories. Red rectangles highlight the greatest level of alloreactivity prediction according the considered model for a given pair.

We aimed to explore if the heterogeneity of the models could be responsible for literature discrepancies regarding the benefit of predicted NK alloreactivity. We first focused on the distribution of alloreactivity according to the 27 models described. **Table 1** shows the distribution of pairs predicted to be alloreactive according to each model within the genoidentical and the haploidentical cohorts. It highlights that the distribution of alloreactivity differs according to the different subset models of each scoring category based on education, KIR haplotypes as well as KIR polymorphisms. Focusing on the ligand/ligand and receptor/ligand categories, the distributions may appear consistent with each other (e.g. Bw4/Bw6 and BwIPD have similar predictions of alloreactivity). **Figure 4** however highlights that the prediction of alloreactivity differs according to the models in those two later categories. Indeed, the pairs contributing to the statistics are different from one model to another.

**Table 1:** Distribution of pairs predicted to be alloreactive according each model.

	Genoidentical N=43 (55,1%)		Haploidentical N=35 (44,9%)		p*
	N	%/mean	N	%/mean	
LIGAND-LIGAND					
**Bw4/Bw6**					0,0004
HVG mismatch	0	0,0	6	17,1	
No mismatch	43	100,0	26	74,3	
GVH mismatch	0	0,0	3	8,6	
**Bw IPD**					0,0025
HVG mismatch	0	0,0	4	11,4	
No mismatch	43	100,0	28	80,0	
GVH mismatch	0	0,0	3	8,6	
**C1/C2**					<0,0001
HVG mismatch	0	0,0	4	11,4	
No mismatch	43	100,0	24	68,6	
GVH mismatch	0	0,0	7	20,0	
**L/L synthesis**					<0,0001
HVG mismatch in at least one model	0	0,0	10	28,6	
No mismatch in any model	43	100,0	14	40,0	
GVH mismatch in at least one model	0	0,0	10	28,6	
HVG mismatch + GVH mismatch	0	0,0	1	2,9	
RECEPTOR-LIGAND					
**2DL1-C2**					
No	35	81,4	21	60,0	0,0368
Yes	8	18,6	14	40,0	
**3DL1-Bw4**					
No	29	67,4	30	85,7	0,0615
Yes	14	32,6	5	14,3	
**3DL2-A3/A11**					0,9440
No	20	46,5	16	45,7	
Yes	23	53,5	19	54,3	
**2DL2/3-C1**					0,5287
No	38	88,4	29	82,9	
Yes	5	11,6	6	17,1	
**R-L quantitative synthesis**					0,2891
0	8	18,6	6	17,1	
1	20	46,5	17	48,6	
2	15	34,9	9	25,7	
3	0	0,0	3	8,6	
**R-L qualitative synthesis**					0,8671
No	8	18,6	6	17,1	
Yes	35	81,4	29	82,9	
EDUCATIONAL					
**Educ-2DL1**					0,0088
Uneducated (without 2DL1)	10 (2)	23,3	14 (2)	40,0	
No predicted alloreactivity	33	76,7	17	48,6	
Predicted alloreactivity	0	0,0	4	11,4	
**Educ-2DL2/3**					0,1853
Uneducated	5	11,6	4	11,4	
No predicted alloreactivity	38	88,4	28	80,0	
Predicted alloreactivity	0	0,0	3	8,6	
**Educ-3DL1**					0,1465
Uneducated (without 3DL1)	17 (3)	39,5	8 (2)	22,9	
No predicted alloreactivity	26	60,5	26	74,3	
Predicted alloreactivity	0	0,0	1	2,9	
**Educ-3DL2**					0,1298
Uneducated	23	53,5	24	68,6	
No predicted alloreactivity	20	46,5	10	28,6	
Predicted alloreactivity	0	0,0	1	2,9	
**Educ-DL synthesis**					0,0004
0	43	100,0	26	74,3	
1	0	0,0	9	25,7	
**Educ-2DS1**					0,2627
No predicted alloreactivity	34	79,1	31	88,6	
Predicted alloreactivity	9	20,9	4	11,4	
DONORS’ HAPLOTYPES					
**B-pattern**					0,9609
0	11	25,6	11	31,4	
1	18	41,9	12	34,3	
2	9	20,9	8	22,9	
3	4	9,3	3	8,6	
4	1	2,3	1	2,9	
**Cen-B**					0,7159
0	17	39,5	14	40,0	
1	19	44,2	13	37,1	
2	7	16,3	8	22,9	
**Tel-B**					0,8051
0	27	62,8	25	71,4	
1	13	30,2	8	22,9	
2	3	7,0	2	5,7	
**KIR-B content score**					0,6608
Neutral	29	67,4	23	65,7	
Better	7	16,3	4	11,4	
Best	7	16,3	8	22,9	
GENE-GENE					
**Donor’s aKIR**					0,4623
0 aKIR	5	11,6	5	14,3	
1 or 2 aKIR	15	34,9	16	45,7	
Greater/equal 3 aKIR	23	53,5	14	40,0	
**R-D aKIR**					0,8593
*Missing*	2	3			
No. of recipient’s aKIR > No of donor’s aKIR	11	26,8	10	31,3	
No. of recipient’s aKIR = No of donor’s aKIR	21	51,2	16	50,0	
Donor has 1 or 2 aKIR more than recipient	4	9,8	4	12,5	
Donor has ≥ 3 aKIR more than recipient	5	12,2	2	6,3	
**Donor’s iKIR**					0,9094
2 or 3 iKIR	13	30,2	11	31,4	
Greater/equal 4 iKIR	30	69,8	24	68,6	
**R-D iKIR**					0,5071
*Missing*	2	3			
No. of recipient’s iKIR > No of donor’s iKIR	13	31,7	11	34,4	
No. of recipient’s iKIR = No of donor’s iKIR	17	41,5	16	50,0	
Donor has 1 or 2 iKIR more than recipient	11	26,8	5	15,6	
ALLELIC POLYMORPHISM					
**3DL1 expression**					0,7294
*Missing*	1		5		
Null or absent	5	11,9	3	10,0	
Low	5	11,9	6	20,0	
High	32	76,2	21	70,0	
**2DL1 R/C dimorphism**					0,8199
Missing	0		4		
No R allele	6	14,0	5	16,1	
At least one R allele	34	79,1	25	80,6	
Ambiguous	3	7,0	1	3,2	
**-21 B dimorphism**					0,9455
Donor TT	24	55,8	21	60,0	
Donor MT	14	32,6	11	31,4	
Donor MM	5	11,6	3	8,6	

Taken together, these results show that models are not interchangeable and that it is wise to investigate further how they could be correlated in genoidentical and haploidentical matching.

### Correlations between the different models

**Figure 5:**
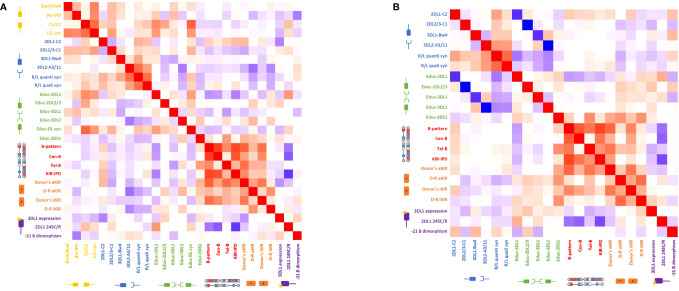
Heatmap of the Spearman’s correlation coefficients reflecting the strength of the association between the different biological models **(A)** for the haploidentical cohort and **(B)** for the genoidentical cohort. Red squares represent a positive correlation between two different models whereas blue squares represent a negative correlation between two different models. See also **Supplementary Tables 1**, **2**.

**Figure 5** represents heatmaps showing the strength of the association between the different biological models explored through Spearman’s correlation coefficients. Because the genoidentical cohort do not allow the assessment of every model, results were primarily explored within the haploidentical cohort (**Figure 5A** and **Supplementary Table 1**), then compared to genoidentical pairs (**Figure 5B** and **Supplementary Table 2**) and across the whole cohort (**Supplementary Figure 1** and **Supplementary Table 3**). Values in brackets below correspond to correlation coefficients.

The most striking correlations are highlighted in the following subsections.

#### Haploidentical cohort

Considering first the KIR haplotype-based categories; (i) KIR-B content score highly correlates with the number of Cen-B but not Tel-B motifs (0.81 and 0.4) as expected regarding the predominant effect of the centromeric part in this scoring strategy; (ii) Tel-B correlates with Educ-2DS1 (0.52), reflecting the fact that KIR2DS1 is only found on the telomeric part of B-haplotypes; (iii) KIR haplotype-based models correlate with the number of donor activating and inhibitory genes – e.g. B-pattern correlates with donor aKIR (0.76) and donor iKIR (0.70). This is related to the B-haplotype architecture, known to have higher gene content and to be especially enriched in aKIR. It is noteworthy that donor aKIR correlates with Tel-B but not Cen-B (0.71 vs 0.50) whereas donor iKIR correlate with Cen-B but not Tel-B (0.60 vs 0.42), reflecting that telomeric part is enriched in aKIR compared to the centromeric part.

Educ-2DL1 inversely correlates with donor KIR haplotype-based models: KIR-B content score (-0.55), B-pattern (-0.48), Cen-B (-0.45), the poorest correlation being for Tel-B (-0.18). This once again highlights the architecture of KIR haplotypes, with KIR2DL1 always present on the A-haplotype but not always on B-haplotypes.

2DL1 245C/R positively correlates with the alloreactivity predicted by Educ-2DL1 (0.55). Consequently, 2DL1 245C/R behaves similarly to the Educ-2DL1 model, i.e. inversely to KIR haplotype-based models. The correlations with B-pattern, Cen-B and KIR-B content score are respectively -0.59, -0.70 and -0.83. There is no correlation between 2DL1 245C/R and Tel-B (0.04). Noting that KIR2DL1 245R is carried by Cen A haplotypes, while 2DL1 245C by Cen B1 haplotypes, this reflects once again the architecture of KIR haplotypes.

#### Genoidentical cohort

Because genoidentical pairs are 10/10 HLA-matched, there is neither D/R ligand-ligand mismatch in any direction, nor predicted alloreactivity within the educational models.

The inverse correlation between the educational models and missing ligand models are much stronger than in the haploidentical cohort, with the following correlation coefficients: 2DL1-C2/Educ-2DL1: -0.87; 3DL1-Bw4/Educ-3DL1: -0.86; 3DL2-A3/11/Educ-3DL1: -1 and 2DL2/3-C1/Educ-2DL2/3: -1. Indeed, the lack of discrepancies between donor and recipient HLA-types allows focus on the KIR-based predicted alloreactivity and on the correlations between those models.

The strengths of correlations between donors’ KIR haplotype-based strategies (B-pattern, Cen-B and KIR-B content score) are similar to the ones observed in the haploidentical cohorts with similar coefficients of correlation. Similar finding are observed for the models based on gene content (donor aKIR, donor iKIR). Indeed, those models only depend on donor KIR genotyping, and the D/R compatibility does not matter. 2DL1 245C/R behaves similarly in both cohorts, i.e. inversely to KIR haplotype-based models with approximately same coefficients of correlations.

### Clinical-biological correlations

We hypothesized that particular models could be differentially predictive of clinical outcomes (death, relapse, aGVHD and cGVHD). Acknowledging the small sizes of the study cohorts as well as their heterogeneity (**Supplementary Tables 4**–**6**), we proceeded to look at the correlations between the different models and recipients’ post-aHSCT outcomes as exploratory results. **Supplementary Table 7** shows the p-values of the Gray test used to compare the cumulative incidence of post-allograft outcomes (death, relapse, aGVHD and cGVHD) according to the results of alloreactivity predictions, death being considered as a competitive risk in the various models.

**Figure 6:**
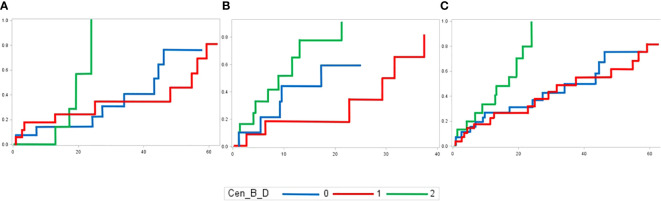
Cumulative incidences of aGVHD in **(A)** genoidentical, **(B)** haploidentical and **(C)** whole cohort according to the number of Cen-B motifs of the donor.

The major clinical-biological correlation is found between the Cen-B model and the cumulative incidence of aGVHD. Higher number of Cen-B is associated with higher cumulative incidence of aGVHD (p-value of 0.08, 0.03 and 0.006 in genoidentical, haploidentical and whole cohort, respectively) (**Figure 6**). Because this effect is found in the genoidentical and in the haploidentical cohort as well, this might suggest that KIR predicted alloreactivity but not MHC mismatches are responsible for aGVHD occurrence in these series. The aGVHD took on average one month to occur after the transplantation (**Supplementary Table 6**), i.e. could not be led by T-lymphocytes resulting from *de novo* repertoire, but from donor’s mature T-lymphocytes present within the graft likely supported by alloreactive donor’s NK cells

## Discussion

We hypothesized that the various models used to predict NK alloreactivity could be partly responsible for published discrepancies. Many models have been described, but they target different basic aspects of the complex biological process of education. The main objective of this work was to examine the heterogeneity of the major KIR-based prediction models in alloreactivity and to assess potential correlations.

Considering first the distributions of predicted alloreactivities among the models, we highlighted that (i) some models cannot be used within genoidentical pairs: indeed, genoidentical donors are 10/10 HLA-matched with the recipients, so there is neither mismatch according to ligand-ligand models nor predicted alloreactivity according to the educational models for those D/R pairs. (ii) Ligand-ligand and education models predict alloreactivity for only a small subset of recipients. Consequently, the stratification of potential donors using these models could have only a moderate benefit. Studies aiming to describe the effect of alloreactivity according to these models will need to enroll a sufficient number of patients. (iii) More importantly, prediction of alloreactivity according different models was not consistent within the same pair.

We then focused on correlations between the models in view of biological relationships. Indeed (i) receptor-ligand models negatively correlate with educational models, especially in the genoidentical cohort where the coefficients’ correlations are about -1. In educational models, a donor’s NK cells are supposed to be alloreactive when the donor has KIR and its cognate MHC molecule, but the recipient lacks the same cognate MHC molecule: this cannot occur in 10/10 HLA matched settings. Alloreactivity according to the receptor-ligand model is counted when a recipient lacks the cognate MHC for a donor KIR, so genoidentical pairs can be predicted as alloreactive according to this model. Because they rely on the same KIR/MHC interactions (i.e. 2DL1-C2, 2DL2/3-C1, 3DL1-Bw4 and 3DL2-A3/A11), they strongly correlate but in opposite directions. (ii) Predicted alloreactivity according to C2-2DL1 education tends to inversely correlate with each model assessing the presence of centromeric B-motifs but not with telomeric B-motif, highlighting the architecture of KIR haplotypes, with *KIR2DL1* always present on A-haplotype but not always on B-haplotypes and the hotspot recombination between centromeric and telomeric parts disrupting the linkage disequilibrium between *KIR2DL1* and telomeric B-motifs. (iii) KIR haplotype-based models positively correlate with the number of donors’ activating and inhibitory genes, reflecting the higher gene content in B-haplotypes compared to A-haplotype. (iv) The presence of *KIR2DL1* 245R allele in donor correlating with 2DL1-C2 education could be explained by *KIR2DL1* copy number variation: the more *KIR2DL1* alleles a donor has, the higher the probability of him carrying at least one 245R allele and fulfilling education. (v) We also highlighted that the correlations between the models may differ according to the D/R compatibility, as the inverse correlations between the educational models and receptor-ligand models are much stronger in the genoidentical cohort compared to the haploidentical cohort.

We finally aimed to explore correlations between the different models and clinical outcomes. We acknowledge that our two cohorts significantly differ regarding recipient characteristics. Despite these limitations, we highlighted the following trends: (i) each model is differentially associated with different outcomes, (ii) the different models predict one outcome with different efficacy and (iii) the D/R compatibility greatly impacts the pertinence of the model.

Among the 27 models, the number of centromeric B-motifs is the only parameter correlated within both cohorts for the same outcome, i.e. the cumulative incidence of aGVHD. Because this effect is found in the genoidentical and the haploidentical cohort, this might suggest that KIR predicted alloreactivity but not MHC mismatching could be responsible for it. Even if the original study describing KIR haplotypes has correlated the presence of centromeric B-motifs with better long-term outcomes (increased overall survival and disease-free survival but decreased relapse) (30), our results should not be interpreted as standing in contradiction with the latter because the shortness of recipients’ follow-up (median of 23 months *vs* 13 months for genoidentical and haploidentical cohorts respectively, **Supplementary Table 6**). However, our results are consistent with a more recent study of a Japanese cohort showing that Bx donors were associated with a higher risk for grade III to IV aGVHD (38). The presence of activating KIR genes on the centromeric B-motif, for example *KIR2DS2*, *KIR2DS3* as well as the *KIR2DL1* 245C allele, could explain this greater alloreactivity. To our best knowledge, this paper is the first to experimentally compare NK alloreactivity prediction models within a cohort of genoidentical and haploidentical aHSCT donor-recipient pairs. This study helps to resolve current discrepancies in KIR-based alloreactivity predictions and highlights the need for deeper consideration of the models used in clinical studies as well as in medical practice.

## Data availability statement

The raw data supporting the conclusions of this article will be made available by the authors, without undue reservation.

## Ethics statement

The studies involving human participants were reviewed and approved by Ethics committee of Nancy’s Centre Hospitalier Régional Universitaire, France Hospital. The patients/participants provided their written informed consent to participate in this study.

## Author contributions

AD, AA and JaT designed the research project. AD was in charge of the establishment and declaration of the biocollection, samples’ preparation as well as their shipping, HLA/KIR typing as well as clinical data management, models’ interpretation and writing of the manuscript. AA extracted the DNA from dried PBMC sent by CRYOSTEM. AA and JaT supported every step of the models’ analyses and interpretation of statistical results. TR accompanied the methodological aspects of the research and performed the statistical analyses. SC performed PCR-SSO for KIR typing. AC provided the written consent for all haploidentical donors. AA, JaT, JoT, TR, MP, VC-E and NN-G reviewed the manuscript. All authors contributed to the article and approved the submitted version.

## Supplementary material

Supplementary Table 1 Spearman’s correlation coefficients between the different biological models within the haploidentical cohort.

Supplementary Table 2 Spearman’s correlation coefficients between the different biological models within the genoidentical cohort.

Supplementary Table 3 Spearman’s correlation coefficients between the different biological models within the whole cohort (genoidentical and haploidentical).

Supplementary Table 4 Recipients’ characteristics per cohort.

Supplementary Table 5 Graft parameters per cohort.

Supplementary Table 6 Recipient’s post-allograft outcomes per cohort.

Supplementary Table 7 p-values of the Gray test used to compare the cumulative incidence of post-allograft’s outcomes (death, relapse, aGVHD and cGVHD) according to the results of alloreactivity predictions’, death being considered as a competitive risk in the various models.

Supplementary Figure 1 Heatmap of the Spearman’s correlation coefficients reflecting the strength of the association between the different biological within the whole cohort. Red squares represent a positive correlation between two different models whereas blue squares represent a negative correlation between two different models. See also **Supplementary Table 3**.

## References

[1] Duarte RF Labopin M Bader P Basak GW Bonini C Chabannon C. Indications for haematopoietic stem cell transplantation for haematological diseases, solid tumours and immune disorders: Current practice in Europe, 2019. Bone Marrow Transplant (2019) 54:1525–52. doi: 10.1038/s41409-019-0516-2 30953028

[2] Kwon M Bailén R Díez-Martín JL. Evolution of the role of haploidentical stem cell transplantation: past, present, and future. Expert Rev Hematol (2020) 13:835–50. doi: 10.1080/17474086.2020.1796621 32749913

[3] Passweg JR Baldomero H Bader P Bonini C Duarte RF Dufour C. Use of haploidentical stem cell transplantation continues to increase: The 2015 European society for blood and marrow transplant activity survey report. Bone Marrow Transplant (2017) 52:811–7. doi: 10.1038/bmt.2017.34 PMC5467246 28287639

[4] Passweg JR Baldomero H Chabannon C Basak GW de la Cámara R Corbacioglu S. Hematopoietic cell transplantation and cellular therapy survey of the EBMT: monitoring of activities and trends over 30 years. Bone Marrow Transplant (2021) 56:1651–64. doi: 10.1038/s41409-021-01227-8 PMC8263343 33623153

[5] Fuchs EJ. Haploidentical transplantation for hematologic malignancies: Where do we stand? Hematol Am Soc Hematol Educ Program (2012) 2012:230–6. doi: 10.1182/asheducation-2012.1.230 PMC3657706 23233586

[6] Spits H Bernink JH Lanier L. NK cells and type 1 innate lymphoid cells: Partners in host defense. Nat Immunol (2016) 17:758–64. doi: 10.1038/ni.3482 27328005

[7] Locatelli F Pende D Falco M Della Chiesa M Moretta A Moretta L. NK cells mediate a crucial graft-versus-Leukemia effect in haploidentical-HSCT to cure high-risk acute leukemia. Trends Immunol (2018) 39:577–90. doi: 10.1016/j.it.2018.04.009 29793748

[8] Moretta A Bottino C Vitale M Pende D Biassoni R Mingari MC. Receptors for hla class-i molecules in human natural killer cells. Annu Rev Immunol (1996) 14:619–48. doi: 10.1146/annurev.immunol.14.1.619 8717527

[9] Yokoyama WM Kim S. Licensing of natural killer cells by self-major histocompatibility complex class I. Immunol Rev (2006) 214:143–54. doi: 10.1111/j.1600-065X.2006.00458.x 17100882

[10] Ljunggren HG Kärre K. In search of the “missing self”: MHC molecules and NK cell recognition. Immunol Today (1990) 11:237–44. doi: 10.1016/0167-5699(90)90097-S 2201309

[11] Russo A Oliveira G Berglund S Greco R Gambacorta V Cieri N. NK cell recovery after haploidentical HSCT with posttransplant cyclophosphamide: dynamics and clinical implications. Blood (2018) 131:247–62. doi: 10.1182/blood-2017-05-780668 PMC5757695 28986344

[12] Savani BN Mielke S Adams S Uribe M Rezvani K Yong ASM. Rapid natural killer cell recovery determines outcome after T-cell-depleted HLA-identical stem cell transplantation in patients with myeloid leukemias but not with acute lymphoblastic leukemia. Leukemia (2007) 21:2145–52. doi: 10.1038/sj.leu.2404892 17673900

[13] Ruggeri L Capanni M Urbani E Perruccio K Shlomchik WD Tosti A. Effectiveness of donor natural killer cell alloreactivity in mismatched hematopoietic transplants. Science (2002) 295:2097–100. doi: 10.1126/science.1068440 11896281

[14] Shah NN Baird K Delbrook CP Fleisher TA Kohler ME Rampertaap S. Acute GVHD in patients receiving IL-15/4-1BBL activated NK cells following t-cell–depleted stem cell transplantation. Blood (2015) 125:784–92. doi: 10.1182/blood-2014-07-592881 PMC4311226 25452614

[15] Ghayur T Seemayer TA Kongshavn PA Gartner JG Lapp WS. Graft-versus-host reactions in the beige mouse. An investigation of the role of host and donor natural killer cells in the pathogenesis of graft-versus-host disease. Transplantation (1987) 44:261–7. doi: 10.1097/00007890-198708000-00017 3307050

[16] Dhuyser A Aarnink A Pérès M Jayaraman J Nemat-Gorgani N Rubio MT. KIR in allogeneic hematopoietic stem cell transplantation: Need for a unified paradigm for donor selection. Front Immunol (2022) 13. doi: 10.3389/fimmu.2022.821533 PMC8886110 35242134

[17] Huang X-J Zhao X-Y Liu D-H Liu K-Y Xu L-P. Deleterious effects of KIR ligand incompatibility on clinical outcomes in haploidentical hematopoietic stem cell transplantation without *in vitro* T-cell depletion. Leukemia (2007) 21:848–51. doi: 10.1038/sj.leu.2404566 17268518

[18] Ruggeri L Capanni M Casucci M Volpi I Tosti A Perruccio K. Role of natural killer cell alloreactivity in HLA-mismatched hematopoietic stem cell transplantation. Blood (1999) 94:333–9. doi: 10.1182/blood.V94.1.333.413a31_333_339 10381530

[19] Wagner I Schefzyk D Pruschke J Schöfl G Schöne B Gruber N. Allele-level KIR genotyping of more than a million samples: Workflow, algorithm, and observations. Front Immunol (2018) 9:2843. doi: 10.3389/fimmu.2018.02843 30564239 PMC6288436

[20] Lange V Böhme I Hofmann J Lang K Sauter J Schöne B. Cost-efficient high-throughput HLA typing by MiSeq amplicon sequencing. BMC Genomics (2014) 15:63. doi: 10.1186/1471-2164-15-63 24460756 PMC3909933

[21] Maiers M Gragert L Klitz W. High-resolution HLA alleles and haplotypes in the united states population. Hum Immunol (2007) 68:779–88. doi: 10.1016/j.humimm.2007.04.005 17869653

[22] Jayaraman J Kirgizova V Di D Johnson C Jiang W Traherne JA. qKAT: Quantitative semi-automated typing of killer-cell immunoglobulin-like receptor genes. J Vis Exp (2019). doi: 10.3791/58646 PMC6794157 30907867

[23] IPD-KIR database. Available at: https://www.ebi.ac.uk/ipd/kir/matching/ligand/ (Accessed November 18, 2022).

[24] Leung W Iyengar R Turner V Lang P Bader P Conn P. Determinants of antileukemia effects of allogeneic NK cells. J Immunol (2004) 172:644–50. doi: 10.4049/jimmunol.172.1.644 14688377

[25] Nowak J Kościńska K Mika-Witkowska R Rogatko-Koroś M Mizia S Jaskuła E. Donor NK cell licensing in control of malignancy in hematopoietic stem cell transplant recipients. Am J Hematol (2014) 89:E176–183. doi: 10.1002/ajh.23802 25044365

[26] Nowak J Kościńska K Mika-Witkowska R Rogatko-Koroś M Mizia S Jaskuła E. Role of donor activating KIR-HLA ligand-mediated NK cell education status in control of malignancy in hematopoietic cell transplant recipients. Biol Blood Marrow Transplant (2015) 21:829–39. doi: 10.1016/j.bbmt.2015.01.018 25617806

[27] Pende D Marcenaro S Falco M Martini S Bernardo ME Montagna D. Anti-leukemia activity of alloreactive NK cells in KIR ligand-mismatched haploidentical HSCT for pediatric patients: Evaluation of the functional role of activating KIR and redefinition of inhibitory KIR specificity. Blood (2009) 113:3119–29. doi: 10.1182/blood-2008-06-164103 18945967

[28] Venstrom JM Pittari G Gooley TA Chewning JH Spellman S Haagenson M. HLA-C–dependent prevention of leukemia relapse by donor activating KIR2DS1. New Engl J Med (2012) 367:805–16. doi: 10.1056/NEJMoa1200503 PMC3767478 22931314

[29] Hsu KC Chida S Geraghty DE Dupont B. The killer cell immunoglobulin-like receptor (KIR) genomic region: Gene-order, haplotypes and allelic polymorphism. Immunol Rev (2002) 190:40–52. doi: 10.1034/j.1600-065X.2002.19004.x 12493005

[30] Cooley S Weisdorf DJ Guethlein LA Klein JP Wang T Le CT. Donor selection for natural killer cell receptor genes leads to superior survival after unrelated transplantation for acute myelogenous leukemia. Blood (2010) 116:2411–9. doi: 10.1182/blood-2010-05-283051 PMC2953880 20581313

[31] IPD - KIR sequence database| EBI. Available at: https://www.ebi.ac.uk/ipd/kir/donor_b_content.html (Accessed September 22, 2019).

[32] Shen M Linn Y-C Ren E-C. KIR-HLA profiling shows presence of higher frequencies of strong inhibitory KIR-ligands among prognostically poor risk AML patients. Immunogenetics (2016) 68:133–44. doi: 10.1007/s00251-015-0888-4 26649563

[33] Boudreau JE Giglio F Gooley TA Stevenson PA Le Luduec J-B Shaffer BC. KIR3DL1/ HL a-b subtypes govern acute myelogenous leukemia relapse after hematopoietic cell transplantation. J Clin Oncol (2017) 35:2268–78. doi: 10.1200/JCO.2016.70.7059 PMC5501362 28520526

[34] Bari R Bell T Leung W-H Vong QP Chan WK Das Gupta N. Significant functional heterogeneity among KIR2DL1 alleles and a pivotal role of arginine245. Blood (2009) 114:5182–90. doi: 10.1182/blood-2009-07-231977 PMC2792213 19828694

[35] Hallner A Bernson E Hussein BA Ewald Sander F Brune M Aurelius J. The HLA-b –21 dimorphism impacts on NK cell education and clinical outcome of immunotherapy in acute myeloid leukemia. Blood (2019) 133:1479–88. doi: 10.1182/blood-2018-09-874990 PMC6440292 30647027

[36] Jagasia MH Greinix HT Arora M Williams KM Wolff D Cowen EW. National institutes of health consensus development project on criteria for clinical trials in chronic graft-versus-Host disease: I. the 2014 diagnosis and staging working group report. Biol Blood Marrow Transplant (2015) 21:389–401.e1. doi: 10.1016/j.bbmt.2014.12.001 25529383 PMC4329079

[37] Glucksberg H Storb R Fefer A Buckner CD Neiman PE Clift RA. Clinical manifestations of graft-versus-host disease in human recipients of marrow from HL-a-matched sibling donors. Transplantation (1974) 18:295–304. doi: 10.1097/00007890-197410000-00001 4153799

[38] Hosokai R Masuko M Shibasaki Y Saitoh A Furukawa T Imai C. Donor killer immunoglobulin-like receptor haplotype b/x induces severe acute graft-versus-Host disease in the presence of human leukocyte antigen mismatch in T cell-replete hematopoietic cell transplantation. Biol Blood Marrow Transplant (2017) 23:606–11. doi: 10.1016/j.bbmt.2016.12.638 28042021

