# Supplementary material for: Comparison of NK alloreactivity prediction models based on KIR-MHC interactions in haematopoietic stem cell transplantation
Source: Front Immunol. 2023 Mar 2;14:1028162. doi: 10.3389/fimmu.2023.1028162 (PMC10017772; doi:10.3389/fimmu.2023.1028162)
Supplement: Supplementary file 1 [file DataSheet_1.pdf]

Supplementary Material

1 Materials and methods

1.1 Cohorts

The genoidentical cohort was established from CRYOSTEM, a French national biocollection dedicated to HSCT complications and resulting from the CRYOSTEM network activities bringing together all French transplant units as well as 28 biological resource centers. 50 genoidentical pairs meeting the following inclusion criteria were selected : adult patients (18-50 years old) transplanted in first remission and having received myeloablative conditioning without anti-thymoglobulin. All recipients received a GVHD prophylaxis based on the administration of ciclosporin, either alone or with mycofenolate mofetil or methotrexate Particular attention was paid to recipient post-allograft outcome characteristics in order to constitute homogeneous groups considering the GVHD, leading to the selection of 16 pairs without any sign of GVHD (8 men and 8 women), 16 pairs with aGVHD without cGVHD, 9 pairs with cGVHD without aGVHD and 9 pairs with both cGVHD and aGVHD.

The haploidentical cohort was established from the local biocollection of the histocompatibility laboratory of the Nancy's CHRU, registered to the French research ministry under the number DC-2020-4068. This latter was built up from aHSCT performed in the Hematology Department of Nancy's University Hospital, Lorraine, France. Every recipient and their respective relative donor (for whom DNA were prospectively extracted for medical purposes) were solicited to be included in this biocollection. They agreed by providing a written consent for biological sample and clinical data collection, as well as their use for research purposes including genetics analysis. The 35 first consecutive haploidentical D/R pairs included in the local biocollection were selected for this study (aHSCT undergone between March 2015 and February 2019). All recipients received a GVHD prophylaxis based on the administration of post-transplantation cyclophosphamide (50 mg/kg/day at day 3 and day 4 after the procedure), plus ciclosporine and mycofenolate mofetil from day 5 after the procedure.

7 D/R pairs from the 50 selected from the CRYOSTEM biocollection were excluded because extracted DNA quantity or quality was insufficient to perform any DNA-based assay. 156 subjects were finally enrolled in our study (43 genoidentical and 35 haploidentical pairs). HLA typing at allelic resolution was available for all subjects and DKMS Life Science Lab GmbH provided KIR genotyping for 147 subjects, as 2 samples from the genoidentical and 7 samples from the haploidentical cohort had insufficient DNA quantity to perform NGS. However, the additional PCR-SSO performed for the 35 donors of the haploidentical cohort allowed the determination of presence vs absence of each KIR gene for four supplementary haploidentical donors. Finally, KIR genotyping was missing for only five recipients, two genoidentical and three haploidentical. There were no missing clinical data.

1.2 DNA extraction and sample preparation

Concerning the **genoidentical pairs**, peripheral blood mononuclear cells provided by the CRYOSTEM biobank were shipped as cell pellets with various quantity of nucleated cells (range: 0,525. 10^6 to 18.10^6 cells). DNA extraction and purification were performed using QIAamp DNA Blood Mini Kit (QIAGEN, Courtaboeuf, France).

For the **haploidentical pairs,** DNA was extracted at time of aHSCT for medical purposes using one of the following kit : Nucleon™ BACC3 Genomic DNA Extraction Kit (GE Healthcare Life Sciences, Velizy Villacoublay, France), Genomic DNA reagent (Precision System Science, Dusseldorf, Germany) or NucleoSpin Blood L Vacuum (Macherey-Nagel, Hoerdt, France) on Hamilton Microlab® STAR (Hamilton Company, Courtaboeuf, France). Excess of DNA was then stored as part of the biocollection.

The quality of extraction (for genoidentical pairs) or reassessment of quality after storage (for haploidentical pairs) were performed using NanoDrop™ One (ThermoFisher Scientific, Courtaboeuf, France) before the MHC and KIR typing, with standards-compliant results.

1.3 MHC and KIR genotyping

1.3.1 NGS genotyping

DKMS Life Science Lab GmbH provided allelic genotyping resolution of all 13 KIR genes (*KIR2DL1, KIR2DL2/2DL3, KIR2DL4, KIR2DL5A, KIR2DL5B, KIR2DS1, KIR2DS2, KIR2DS3, KIR2DS3/2DS5, KIR2DS4, KIR3DL1/3DS1, KIR3DL2, KIR3DL3),* 2 KIR pseudogenes (*KIR2DP1* and *-3DP1*) and HLA genes (*HLA-A, -B, -C, -DRB1, -DQA1, -DQB1, -DPA1, -DPB1* and *-DRB3/4/5)* as previously described (16,17). DNA for optimum performances was required to be concentrated at >20ng/µL in >100µL/tube but some samples were shipped at 5ng/µL in 60 µL. DNA concentrations were measured by fluorescence (SYBR Green, Biozym, Hessisch Oldendorf, Germany) using the TECAN infinite 200 Pro (Tecan, Männedorf, Switzerland) plate reader. **KIR genotyping:** Samples with DNA concentrations of <2 ng/µl were excluded from KIR genotyping. Four separate PCR amplifications for targeted exons (exon 4, exon 5, exons 3 and exon 7 multiplexed and one amplicon spanning exons 8 and 9) were performed with FastStart™ Taq DNA Polymerase (Roche, Basel, Switzerland) and the associated buffer system with appropriate volumes to obtain balanced read coverage for each KIR exon. Target-specific primers and index primers were obtained from Metabion (metabion international AG, Planegg, Germany). Secondary PCR was performed on the pool to elongate the amplicons with indexes and sequencing adapters for Illumina sequencing following suppliers' recommendations. Samples were then locus pooled before being pooled all together (384 samples). After purification with SPRI - solid phase reversible immobilization - select beads AMPure XP (BeckmanCoulter, Brea, USA), concentration of the pool was checked via qPCR using an ECO Real-Time PCR cycler (Illumina, San Diego, USA) **HLA genotyping:** After unique indexing and addition of adapter sequences of each sample, PCR for exon 2 and exon 3 amplification was performed on Fluidigm Access Array microfluidic chips, combining 48 samples with 48 primer groups for PCR amplification in 2048 individual 35nL reaction chambers. Following PCR, the 48 samples of one Fluidigm chip were pooled, then pool was purified using SPRI bead technology, AMPure XP (Beckman Coulter, Brea, USA) before being quantified with qPCR. **KIR and HLA pooling:** Commonly, 10 KIR amplicon pools are combined with 10 HLA amplicon pools, then 20 pools are combined to form a NovaSeq pool which was again checked by qPCR. Denaturation and dilution of

the sequencing library were executed as recommended by Illumina with 10% PhiX spiked in. **Yield:** As NovaSeq (Illumina, San Diego, USA) can load approximately 7500 samples per run achieving around 1.000 M reads, each sample had about 100k reads among which 5k to 20k reads assigned to each KIR amplicon and the rest for HLA amplicons. **Bioinformatic tools** will not be detailed here. **Data analysis:** for KIR genotyping, allele differentiation was achieved by sequence variations inside the sequenced exons. Alleles that differ outside of these regions could not be distinguished. Lack of phasing information between the exons could lead to some ambiguities reported as Genotype List Strings (GL Strings). If data quality was insufficient for reliable allele level genotyping, only the presence or the absence of the KIR gene has been reported. Concerning HLA genotyping, allelic resolution was assessed for all samples.

1.3.2 PCR-SSO genotyping

For the **haploidentical pairs**, presence or absence of donor's KIR genes and pseudogenes has also been assessed by PCR-SSO using the Luminex® xMAP® technology. The KIR genotypes were determined with the KIR SSO Genotyping Test (One Lambda, Canoga Park, CA, USA) following the recommendations of the suppliers. The data analysis was performed with Fusion software (One Lambda, Canoga Park, CA, USA) with default parameters.

1.4 Data collection

Collected data concerned (i) donor: gender, (ii) recipient: gender, age at time of transplant, pathology leading to allograft, remission status at time of transplant but also post-allograft outcomes – death, relapse, aGVHD and cGVHD - and (iii) graft's parameters: conditioning regimen, graft's source and sex-mismatch.

The previous were defined as following: considering recipients' features, pathology leading to allograft were split into three categories: acute myeloid leukemia (AML) or myelodysplastic syndrome (MDS) / lymphoma / acute lymphoid leukemia (ALL) or undifferentiated acute leukemia (uAL). A complete remission (CR) for AML/MDS was identified when there was proof of molecular or cytogenic remission. CR for lymphoma was identified if the radiologic evaluation showed a normal TDM (and a normal PET-TDM if lesions were detectable with PET at time of diagnosis). (iii) Considering graft's parameters: recipient's conditioning regimen could be either myeloablative (MAC) or non-myeloablative (NMAC) and graft source could be either bone marrow (BM) or peripheral blood stem cell (PBSC).

2 Supplementary Figures and Tables

2.1 Supplementary Figures

Supplementary figure 1: Heatmap of the Spearman's correlation coefficients reflecting the strength of the association between the different biological within the whole cohort. Red squares represent a positive correlation between two different models whereas blue squares represent a negative correlation between two different models. See also supplementary table 3.

2.2 Supplementary Tables

Supplementary table 1: Spearman's correlation coefficients between the different biological models within the haploidentical cohort

Supplementary table 2: Spearman's correlation coefficients between the different biological models within the genoidentical cohort

Supplementary table 3: Spearman's correlation coefficients between the different biological models within the whole cohort (genoidentical and haploidentical)

Supplementary table 4: Recipients' characteristics per cohort

Supplementary table 5: Graft parameters per cohort

Supplementary table 6: Recipient's post-allograft outcomes per cohort

Supplementary table 7: p-values of the Gray test used to compare the cumulative incidence of post-allograft's outcomes (death, relapse, aGVHD and cGVHD) according to the results of alloreactivity predictions', death being considered as a competitive risk in the various models
