# Supplementary material for: Comparison of NK alloreactivity prediction models based on KIR-MHC interactions in haematopoietic stem cell transplantation
Source: Front Immunol. 2023 Mar 2;14:1028162. doi: 10.3389/fimmu.2023.1028162 (PMC10017772; doi:10.3389/fimmu.2023.1028162)
Supplement: Supplementary Table 4 — Recipients’ characteristics per cohort. [file Table_4.docx]

| Supplementary table 4: Recipients’ characteristics per cohort | | | | | | | | | | | | | | | |
| --- | --- | --- | --- | --- | --- | --- | --- | --- | --- | --- | --- | --- | --- | --- | --- |
|  |  |  | **(i) Genoidentical** | | | | |  | **(ii) Haploidentical** | | | | |  |  |
|  |  |  | N=43 (55,1%) | | | | |  | N=35 (44,9%) | | | | |  |  |
|  |  |  | N |  | %/mean |  | SD* |  | N |  | %/mean |  | SD* |  | p** |
| **Gender** | | | | | | | | | | | | | | | 0,1646 |
|  | Male |  | 19 |  | 44,2 |  |  |  | 21 |  | 60,0 |  |  |  |  |
|  | Female |  | 24 |  | 55,8 |  |  |  | 14 |  | 40,0 |  |  |  |  |
| **Age at time of transplant (years, y.)** | |  | 43 |  | 33,82 |  | 9,85 |  | 35 |  | 44,48 |  | 15,27 |  | **0,0004** |
| **Age at time of transplant (< or > 45y)** | | | | | | | | | | | | | | | **0,0001** |
|  | < 45 y. |  | 38 |  | 88,4 |  |  |  | 17 |  | 48,6 |  |  |  |  |
|  | 45 y. or more |  | 5 |  | 11,6 |  |  |  | 18 |  | 51,4 |  |  |  |  |
| **Pathology requiring aHSCT** | | | | | | | | | | | | | | | **0,0387** |
|  | MDS / AML |  | 21 |  | 48,8 |  |  |  | 19 |  | 54,3 |  |  |  |  |
|  | Lymphoma |  | 20 |  | 46,5 |  |  |  | 9 |  | 25,7 |  |  |  |  |
|  | ALL or undifferenciated AL |  | 2 |  | 4,7 |  |  |  | 7 |  | 20,0 |  |  |  |  |
|  |  |  |  |  |  |  |  |  |  |  |  |  |  |  |  |

* SD : standard deviation

** Chi-2 test or Fisher's exact test for qualitative variables, test from a Student's test for quantitative variables

* median

** Chi-square test or Fisher's exact test for qualitative variables, Wilcoxon test for quantitative variables
