# Supplementary material for: Comparison of NK alloreactivity prediction models based on KIR-MHC interactions in haematopoietic stem cell transplantation
Source: Front Immunol. 2023 Mar 2;14:1028162. doi: 10.3389/fimmu.2023.1028162 (PMC10017772; doi:10.3389/fimmu.2023.1028162)
Supplement: Supplementary Table 5 — Graft parameters per cohort. [file Table_5.docx]

| Supplementary table 5: Graft parameters per cohort | | | | | | | | | | | | | | | | | | | |
| --- | --- | --- | --- | --- | --- | --- | --- | --- | --- | --- | --- | --- | --- | --- | --- | --- | --- | --- | --- |
|  |  |  | **(i) Genoidentical** | | | | | | |  | **(ii) Haploidentical** | | | | | | |  |  |
|  |  |  | N=43 (55,1%) | | | | | | |  | N=35 (44,9%) | | | | | | |  |  |
|  |  |  | N |  | %/med* |  | Min |  | Max |  | N |  | %/med* |  | Min |  | Max |  | p** |
| **Complete remission at time of aHSCT** | | | | | | | | | | | | | | | | | | | **<0,0001** |
|  | No |  | 0 |  | 0,0 |  |  |  |  |  | 20 |  | 57,1 |  |  |  |  |  |  |
|  | Yes |  | 43 |  | 100,0 |  |  |  |  |  | 15 |  | 42,9 |  |  |  |  |  |  |
| **Delay between diag. and aHSCT (months)** | |  | 43 |  | 4,8 |  | 3 |  | 85,8 |  | 35 |  | 7,2 |  | 2,88 |  | 106,9 |  | **0,0027** |
| **Delay between diag. and aHSCT (< ou > 1y)** | | | | | | | | | | | | | | | | | | | **0,0078** |
|  | < 1 y |  | 38 |  | 88,4 |  |  |  |  |  | 22 |  | 62,9 |  |  |  |  |  |  |
|  | 1 y or more |  | 5 |  | 11,6 |  |  |  |  |  | 13 |  | 37,1 |  |  |  |  |  |  |
| **Conditioning regimen** | | | | | | | | | | | | | | | | | | | **<0,0001** |
|  | NMAC |  | 1 |  | 2,3 |  |  |  |  |  | 27 |  | 77,1 |  |  |  |  |  |  |
|  | MAC |  | 42 |  | 97,7 |  |  |  |  |  | 8 |  | 22,9 |  |  |  |  |  |  |
| **Graft source** | | | | | | | | | | | | | | | | | | | **<0,0001** |
|  | PBSC |  | 17 |  | 39,5 |  |  |  |  |  | 31 |  | 88,6 |  |  |  |  |  |  |
|  | BM |  | 26 |  | 60,5 |  |  |  |  |  | 4 |  | 11,4 |  |  |  |  |  |  |
| **Sexe mismatch** | | | | | | | | | | | | | | | | | | | 0,8671 |
|  | Female donor for male recipient |  | 8 |  | 18,6 |  |  |  |  |  | 6 |  | 17,1 |  |  |  |  |  |  |
|  | Others |  | 35 |  | 81,4 |  |  |  |  |  | 29 |  | 82,9 |  |  |  |  |  |  |

* median

** Chi-square test or Fisher's exact test for qualitative variables, Wilcoxon test for quantitative variables
