# Supplementary material for: Comparison of NK alloreactivity prediction models based on KIR-MHC interactions in haematopoietic stem cell transplantation
Source: Front Immunol. 2023 Mar 2;14:1028162. doi: 10.3389/fimmu.2023.1028162 (PMC10017772; doi:10.3389/fimmu.2023.1028162)
Supplement: Supplementary Table 6 — Recipient’s post-allograft outcomes per cohort. [file Table_6.docx]

| Supplementary table 6: recipient’s post-allograft outcomes per cohort | | | | | | | | | | | | | | | | | | | |
| --- | --- | --- | --- | --- | --- | --- | --- | --- | --- | --- | --- | --- | --- | --- | --- | --- | --- | --- | --- |
|  |  |  | **(i) Genoidentical** | | | | | | |  | **(ii) Haploidentical** | | | | | | |  |  |
|  |  |  | N=43 (55,1%) | | | | | | |  | N=35 (44,9%) | | | | | | |  |  |
|  |  |  | N |  | %/med* |  | Min |  | Max |  | N |  | %/med* |  | Min |  | Max |  | p** |
| **Death at time of data collection** | | | | | | | | | | | | | | | | | | | 0,1894 |
|  | No |  | 35 |  | 81,4 |  |  |  |  |  | 24 |  | 68,6 |  |  |  |  |  |  |
|  | Yes |  | 8 |  | 18,6 |  |  |  |  |  | 11 |  | 31,4 |  |  |  |  |  |  |
| **Delay between aHSCT and death (months)** | |  | 8 |  | 27,19 |  | 6,93 |  | 56,41 |  | 11 |  | 3,25 |  | 1,28 |  | 9,07 |  | **0,0036** |
| **Delay between aHSCT and last news (months)** | |  | 35 |  | 23,89 |  | 2,83 |  | 62,69 |  | 24 |  | 13,73 |  | 4,11 |  | 37,42 |  | **0,0111** |
| **Diagnosis of acute GVH** | | | | | | | | | | | | | | | | | | | 0,5982 |
|  | No |  | 21 |  | 48,8 |  |  |  |  |  | 15 |  | 42,9 |  |  |  |  |  |  |
|  | Yes |  | 22 |  | 51,2 |  |  |  |  |  | 20 |  | 57,1 |  |  |  |  |  |  |
| **Delay between aHSCT and 1st diagnosis of aGVH (days)** | |  | 22 |  | 26,00 |  | 11,00 |  | 58,00 |  | 20 |  | 31,00 |  | 17,00 |  | 203,00 |  | 0,2630 |
| **Diagnosis of chronic GVH** | | | | | | | | | | | | | | | | | | | 0,4579 |
|  | No |  | 26 |  | 60,5 |  |  |  |  |  | 24 |  | 68,6 |  |  |  |  |  |  |
|  | Yes |  | 17 |  | 39,5 |  |  |  |  |  | 11 |  | 31,4 |  |  |  |  |  |  |
| **Delay between aHSCT and 1st diagnosis of cGVH (months)** | |  | 16 |  | 6,31 |  | 2,46 |  | 17,84 |  | 10 |  | 6,80 |  | 5,19 |  | 13,50 |  | 0,2570 |
| **Relapse** | | | | | | | | | | | | | | | | | | | 0,8083 |
|  | No |  | 36 |  | 83,7 |  |  |  |  |  | 30 |  | 85,7 |  |  |  |  |  |  |
|  | Yes |  | 7 |  | 16,3 |  |  |  |  |  | 5 |  | 14,3 |  |  |  |  |  |  |
| **Delay between aHSCT and relapse (months)** | |  | 7 |  | 8,15 |  | 3,45 |  | 20,04 |  | 5 |  | 5,13 |  | 1,31 |  | 18,96 |  | 0,4340 |
|  |  |  |  |  |  |  |  |  |  |  |  |  |  |  |  |  |  |  |  |

* median

** Chi-square test or Fisher's exact test for qualitative variables, Wilcoxon test for quantitative variables
