# Supplementary figures and images for: Comparison of NK alloreactivity prediction models based on KIR-MHC interactions in haematopoietic stem cell transplantation
Source: Front Immunol. 2023 Mar 2;14:1028162. doi: 10.3389/fimmu.2023.1028162 (PMC10017772; doi:10.3389/fimmu.2023.1028162)

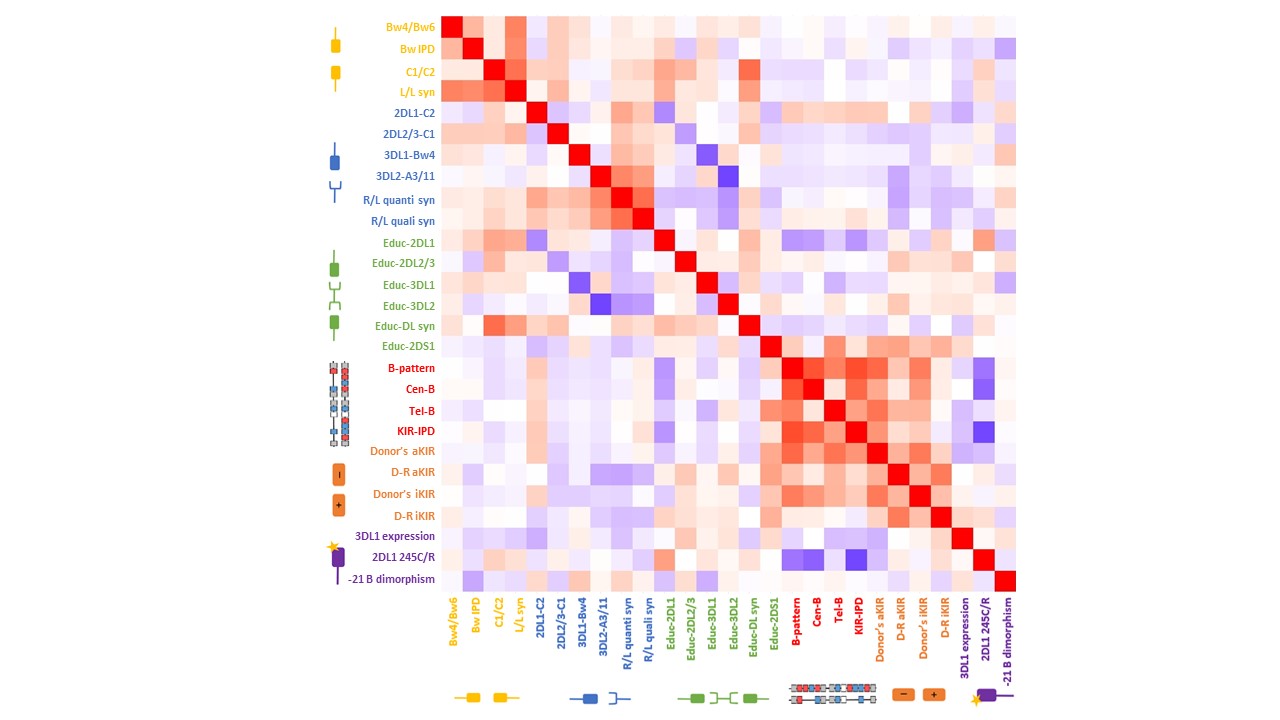

Supplement: Supplementary Figure 1 — Heatmap of the Spearman’s correlation coefficients reflecting the strength of the association between the different biological within the whole cohort. Red squares represent a positive correlation between two different models whereas blue squares represent a negative correlation between two different models. See also Supplementary Table 3. [file Image_1.jpeg]
